# Gaze palsy in glycine receptor antibody-mediated autoimmune encephalitis: a case report

**DOI:** 10.1007/s13760-024-02681-z

**Published:** 2024-11-08

**Authors:** Wan Jiang, Chong Wang, Yun Xu, Qing Ye

**Affiliations:** Department of Neurology, Nanjing Drum Tower Hospital, Affiliated Hospital of Medical School, Nanjing University, 210008 Nanjing, China

## Introduction

Horizontal gaze palsy is traditionally attributed to disturbances in the lateral gaze centers, including the posterior part of the middle frontal gyrus and the paramedian pontine reticular formation (PPRF) [1]. These supranuclear origins typically do not involve the projections from the vestibular nuclei to extraocular motoneurons, thus preserving the vestibulo-ocular reflex [2]. Herein, we describe a rare case of horizontal gaze palsy with a completely impaired vestibulo-ocular reflex, caused by glycine receptor (GlyR) autoantibody.

## Case presentation

**Fig. 1:**
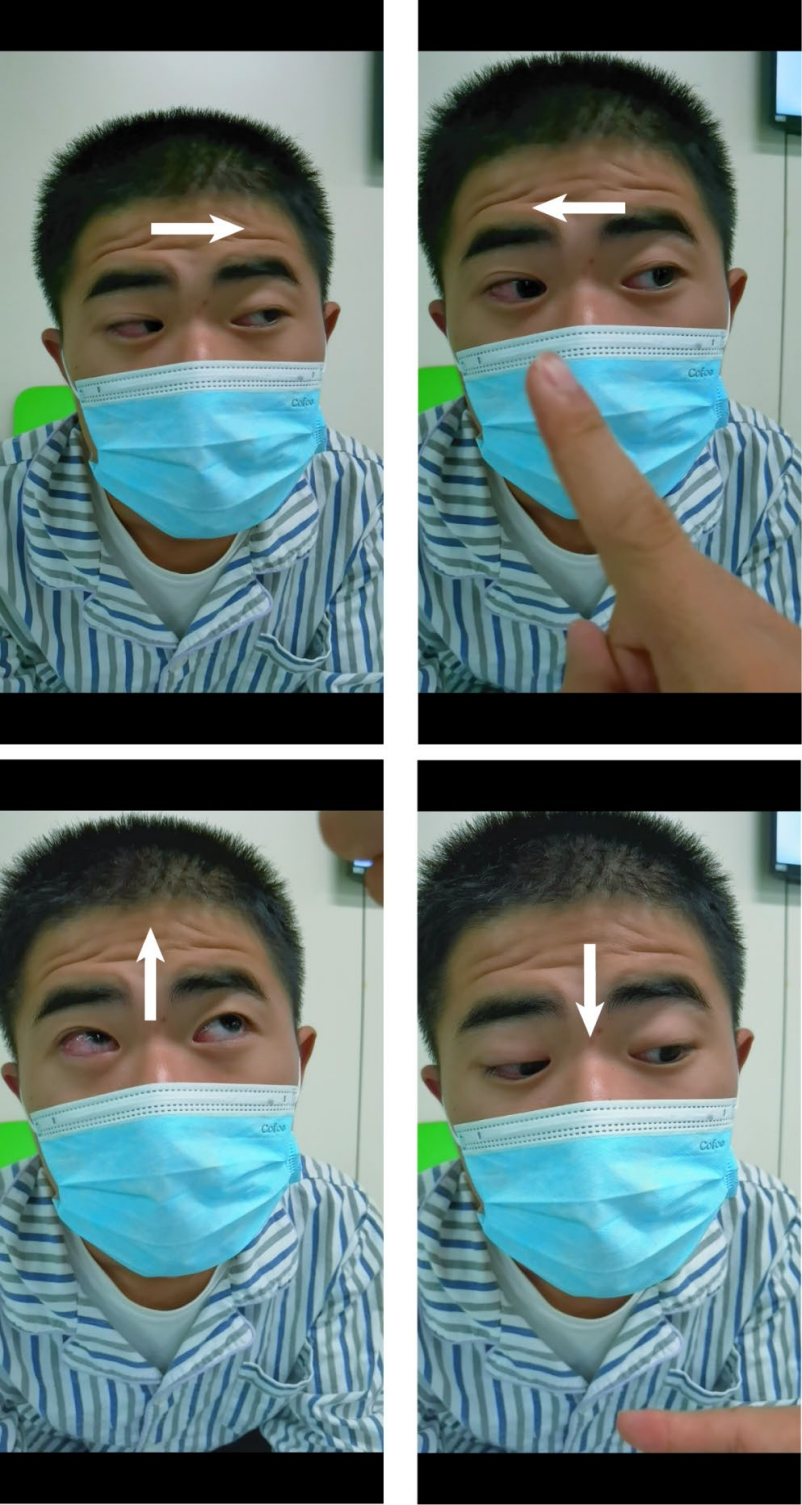
Sustained left gaze was shown by the patient. The eyes could not look to the right or forward, but vertical movement was unrestricted

A 16-year-old male presented with a 7-day history of leftward horizontal gaze palsy. Concurrently, he experienced dizziness during the first 2 days following the onset of gaze palsy, lasting approximately 1–3 h per day. Additionally, he reported a history of fever and diarrhea 20 days prior to the onset of his current symptoms. Physical examination revealed a sustained leftward horizontal gaze; the eyes were immobile in the horizontal plane (even to the forward position), yet vertical movement remained unrestricted (Fig. 1). The vestibulo-ocular reflex was entirely absent, with the eyes appearing “fixed” to the left during the examination (Supplementary Material 1). The patient also exhibited mild left peripheral facial paralysis, while the remainder of the neurological examination was unremarkable. His medical and family histories were non-contributory.

Cerebrospinal fluid (CSF) analysis demonstrated a normal leukocyte count (2 × 10^9^/L) and an increased lymphocyte proportion (83.3%, normal range: 40–80%). Routine electroencephalogram (EEG) was unremarkable, whereas long-term video-EEG revealed episodic spike waves in the bilateral temporal lobes. Brain routine and enhanced magnetic resonance imaging scans were unremarkable (Supplementary Material 2). Antibodies to NMDA, LGI1, IgLON5, GABA, CASPR2, GAD65, and DPPX receptors were negative in both CSF and serum, yet the antibody to alpha1 glycine receptor (GlyR) was positive in both CSF (1:10, cell-based assay) and serum (1:100, cell-based assay).

**Fig. 2:**
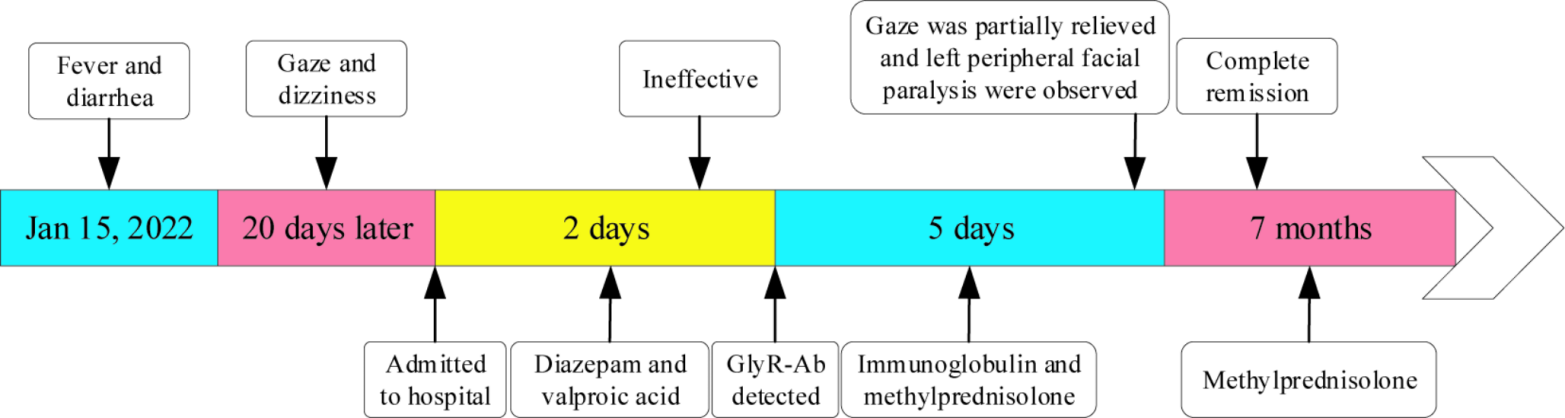
Timeline of the case report. GlyR-Ab, glycine receptor antibody

Diazepam and valproic acid were initiated but failed to alleviate the gaze palsy. Following the receipt of antibody test results, he underwent intravenous immunoglobulin (0.4 g/kg/day) therapy for 5 days. Additionally, intravenous methylprednisolone (500 mg/day for 3 days) was administered, followed by a tapering regimen of 250 mg/day for 3 days, 120 mg/day for 3 days, and methylprednisolone tablets (tapering over approximately 7 months from 60 mg/day to 4 mg/day) (Fig. 2). On the third day of immunotherapy, the gaze palsy partially improved. He could look straight ahead but not to the right. Bilateral horizontal nystagmus and marked left peripheral facial paralysis were also observed. After 10 days of immunotherapy, eye movement fully recovered. The nystagmus and facial paralysis gradually resolved over the subsequent month. Three months post-onset, GlyR antibody titers converted to negative in both CSF and serum.

### Discussion and conclusions

Since 2008, GlyR autoantibody has been identified in several cases of progressive encephalomyelitis with rigidity and myoclonus (PERM), a severe progressive variant of stiff-person syndrome [3, 4]. Patients harboring GlyR autoantibody exhibit complex clinical features, including pronounced hyperekplexia, spasms, rigidity, exaggerated startle, phobias, and brainstem disturbance. Oculomotor disturbances, including gaze palsy, diplopia, ptosis, and nystagmus, are also observed in these patients. Horizontal gaze palsy has been documented in several cases, with patients unable to look to the left, right, or both directions but able to look straight ahead [3, 5, 6]. However, to our knowledge, the sustained horizontal gaze (or “fixed” to one side in the horizontal direction) demonstrated by our patient has not been previously reported. The reason for this may be that gaze palsy is often overshadowed by other clinical presentations in such patients. Traditionally, gaze palsy is associated with supranuclear origins. The absence of the vestibulo-ocular reflex in this case suggests a brainstem lesion. The peripheral facial paralysis also supports the involvement of discrete brainstem areas. Furthermore, the focal involvement and the relatively limited clinical features might be explained by the low titers of GlyR autoantibody.

Both GABAergic and glycinergic projections mediate inhibitory neurotransmission from the vestibular nuclei to the extraocular motoneurons. GABAergic projections predominantly support vertical and oblique extraocular motoneurons, while glycinergic projections primarily support horizontal extraocular motoneurons, including lateral rectus motoneurons and abducens internuclear neurons [7]. Thus, unilateral brainstem involvement by GlyR autoantibody may disrupt inhibitory neurotransmission within the ocular motor system, leading to sustained activation of ipsilateral ocular motoneurons. This could account for the sustained horizontal gaze observed in this case. Moreover, if the lesion involves the PPRF or the sixth abducens nucleus, gaze palsy and the disappearance of the vestibulo-ocular reflex would also ensue.

In conclusion, horizontal gaze palsy may be a clinical feature in patients with GlyR autoantibody, and the vestibulo-ocular reflex is crucial for localization in diagnosis. This case may raise awareness about the rare causes of horizontal gaze palsy.

## Electronic supplementary material

Below is the link to the electronic supplementary material.

Supplementary Material 1

Supplementary Material 2

Supplementary Material 3

## Data Availability

No datasets were generated or analysed during the current study.
