# Supplementary figures and images for: Gaze palsy in glycine receptor antibody-mediated autoimmune encephalitis: a case report
Source: Acta Neurol Belg. 2024 Nov 8;125(3):833–5. doi: 10.1007/s13760-024-02681-z (PMC12126360; doi:10.1007/s13760-024-02681-z)

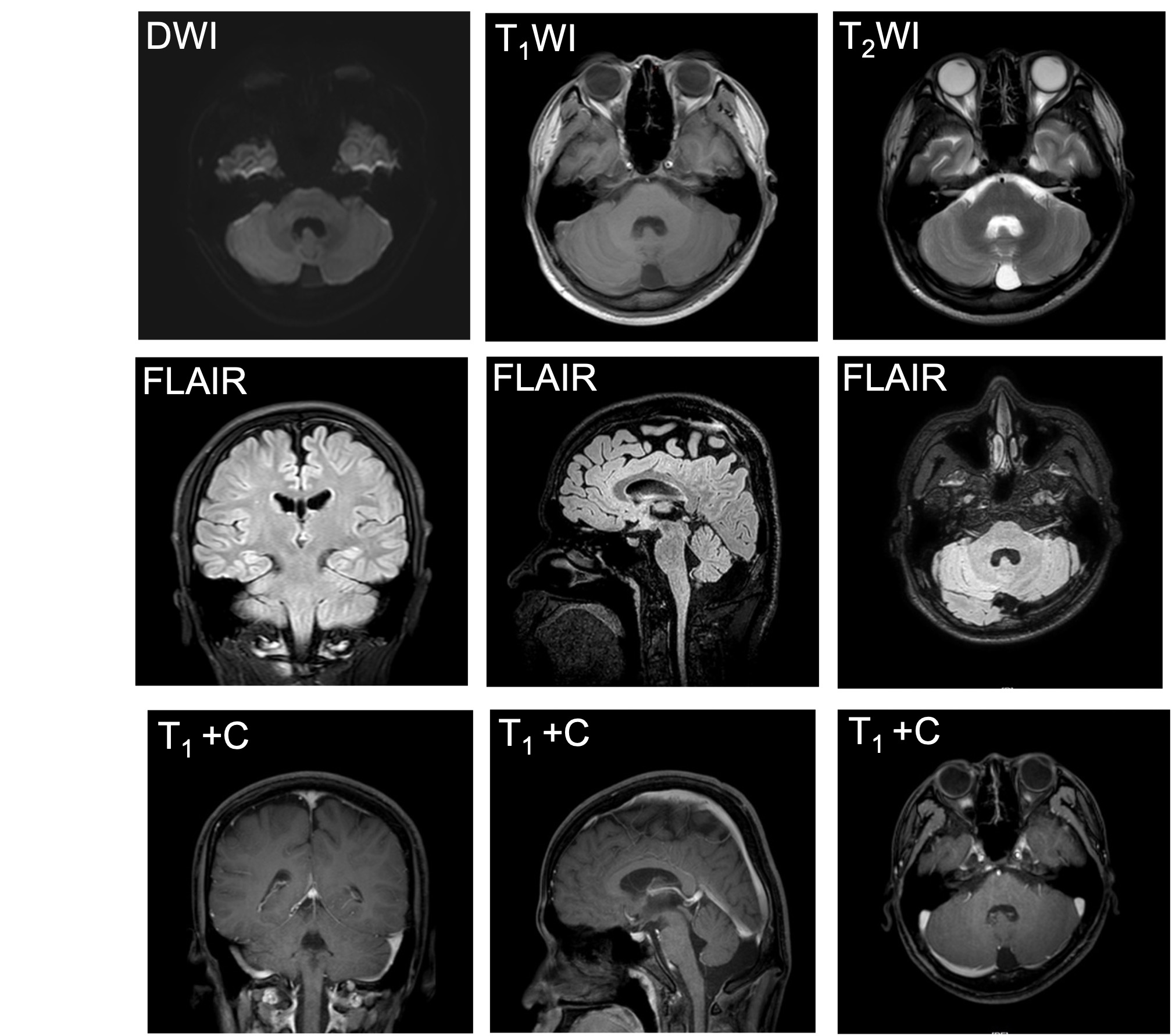

Supplement: Supplementary file 2 — Supplementary Material 2 [file 13760_2024_2681_MOESM2_ESM.tiff]
